# BRD4 Inhibition Protects Against Acute Pancreatitis Through Restoring Impaired Autophagic Flux

**DOI:** 10.3389/fphar.2020.00618

**Published:** 2020-05-08

**Authors:** Shuangjun Shen, Bin Li, Juanjuan Dai, Zengkai Wu, Yan He, Li Wen, Xingpeng Wang, Guoyong Hu

**Affiliations:** ^1^ Department of Gastroenterology, Shanghai General Hospital, Shanghai Jiao Tong University School of Medicine, Shanghai, China; ^2^ Shanghai Key Laboratory of Pancreatic Disease, Institute of Pancreatic Disease, Shanghai Jiao Tong University School of Medicine, Shanghai, China

**Keywords:** BRD4, acute pancreatitis, autophagic flux, SIRT1, lysosomal degradation

## Abstract

Impaired autophagy has been shown to play a critical role in experimental and human acute pancreatitis (AP). However, the mechanism for transcriptional regulation of autophagy remains largely unknown. In this study, we aim to explore the role of BRD4 (bromodomain-containing protein 4), a transcriptional repressor of autophagy, during AP. Changes in pancreatic BRD4 expression and the effect of BRD4 inhibition were measured in mice with AP (induced by caerulein and ethanol and palmitoleic acid) and in isolated pancreatic acinar cells stimulated with cholecystokinin (CCK). Pancreatitis severity was evaluated by serum amylase and pancreatic histopathology. The autophagic flux, the fusion of autophagosome and lysosome, and lysosomal degradation were evaluated. Sirtuin 1 (SIRT1) expression and the effect of SIRT1 inhibition were assessed. We found that pancreatic BRD4 expression was upregulated during various models of AP. BRD4 inhibition reduced CCK-stimulated pancreatic acinar cell injury and pro-inflammatory expression *in vitro* and protected against two models of experimental AP. Mechanistically, BRD4 inhibition restored impaired autophagic flux *via* promoting autophagosome-lysosome fusion and lysosomal degradation. BRD4 inhibition also upregulated SIRT1 and inhibition of SIRT1 reversed the effects of BRD4 inhibition on autophagic flux. Our data suggest that BRD4 is a potential therapeutic target for treating AP.

## Introduction

Acute pancreatitis (AP) is an inflammatory disease of the exocrine pancreas, which is closely related to high morbidity and mortality, and has an increasing incidence in recent years worldwide (Pandol et al., 2007; Habtezion et al., 2019). Currently, there is no effective treatment that can change the pathological process of the disease (Vege et al., 2018). Autophagy is an essential cellular process which can degrade defective cellular proteins and recycle useful components (Parzych and Klionsky, 2014). In experimental pancreatitis, impaired autophagy is due to the disability of lysosomes to degrade cargo and a consequent increase in the formation of autophagosomes (Gukovskaya et al., 2017; Habtezion et al., 2019). Studies focusing on autophagy related genes knockout mice models revealed the mechanistic role of autophagy in maintaining the homeostasis of pancreas and the pathogenesis of AP. Autophagy genes (Atg) 5 and Atg7 knockout mice developed pancreatitis and extensive fibrosis (Antonucci et al., 2015; Diakopoulos et al., 2015). In addition, the impaired lysosomal function, for example in lysosomal-associated membrane protein 2 (LAMP2) deficient mice, leading to pancreatic inflammation and acinar cell degeneration. Furthermore, LAMP2 deficiency also increased the severity of experimental pancreatitis (Mareninova et al., 2015). All these findings showed the essential role of impaired autophagy in the development of AP, suggesting pharmacologic approaches to enhance autophagy efficiency may be of great importance to treat AP.

Bromodomain-containing protein 4 (BRD4) is a member of the Bromo- and Extra-Terminal domain (BET) family proteins with two N-terminal bromodomains and an extra-terminal domain (Shi and Vakoc, 2014; Hogg et al., 2017). BRD4 plays a crucial role in regulating gene transcription during cellular proliferation and differentiation processes (Shi and Vakoc, 2014; Jiang et al., 2017). Moreover, BRD4 has been recently identified to repress autophagy and lysosomal genes transcription by binding to the promoter regions (Sakamaki et al., 2017). BRD4 inhibition can enhance autophagic flux and lysosomal function, thus promoting the degradation of pathogenic proteins (Sakamaki et al., 2017). However, the role of BRD4 in the pathogenesis of AP remains undetermined.

Therefore, we aim to explore the role of BRD4 using experimental models of pancreatitis. Firstly, we found that BRD4 is upregulated in various models of AP. Inhibition of BRD4 by JQ1 protected against pancreatic acinar cell injury induced by cholecystokinin (CCK). Furthermore, inhibition or knockdown of BRD4 restored impaired autophagic flux *via* enhancing autophagosome-lysosome fusion and lysosomal degradation. Interestingly, BRD4 did not alter the initiation of autophagy in pancreatic acinar cells. In addition, BRD4 inhibition upregulated SIRT1 and inhibition of SIRT1 reversed the effects of BRD4 inhibition on autophagic flux, suggesting that inhibition of BRD4 *via* upregulating SIRT1 exerted its effects on autophagy. Finally, we showed that BRD4 inhibition also protected against two clinically representative models of AP through restoring impaired autophagy *in vivo*.

## Materials and Methods

### Chemicals and Reagents

Caerulein (CER) (#HY-A0190) was purchased from MedChemExpress (Monmouth Junction, NJ). Palmitoleic acid (POA), CCK, L-Arginine (L-Arg), and Na taurocholate (NaT) were purchased from Sigma-Aldrich Chemical (St. Louis, MO, USA). BSA was purchased from Yeasen (Shanghai, China). JQ1, EX527 were purchased from Selleck Chemicals (Houston, TX, USA). Antibodies against microtubule associated protein 1 light chain 3 beta (LC3B) (#3868S), p62 (#23214S), ATG14 (#96752S), cathepsin B (#31718S), AMP activated protein kinase (AMPK) (#5831T), phosphorylated AMPK (#2535T), mammalian target of rapamycin (mTOR) (#2983T), phosphorylated mTOR (#5536T), β-actin (#3700s) were purchased from Cell Signaling Technology (Danvers, MA, USA). Antibody against Amylase (#sc46657) were purchased from Santa Cruz Biotechnology (Dallas, TX, USA). Antibodies against BRD4 (#ab128874), LAMP2 (#ab203224), syntaxin 17 (STX17) (#ab229646), cathepsin L (#ab6314) were purchased from Abcam (Cambridge, MA, USA). Antibody against CD45 (#550539) were purchased from BD Biosciences (Franklin, NJ, USA).

### Animals

All experiments involving animals were approved by the Institutional Animal Care and Research Ethics Committee of Shanghai Jiao Tong University School of Medicine (SYXK 2013–0050, Shanghai, China) and carried out in accordance with the guidelines of the National Institute of Health for the Care and Use of Laboratory Animals. Male C57BL/6 mice (6–8 weeks, 20–22 g) were purchased from Shanghai SLAC Laboratory Animal Co Ltd (Shanghai, China). All mice were housed under specific pathogen-free environment with controlled temperature (23 ± 1°C), 12 h light-dark cycle, humidity of 40–70% and free access to water and standard rodent diet. Mice were randomly allocated into groups for all the *in vivo* studies (n = 5 per group).

### Isolation and Treatments of Mouse Pancreatic Acinar Cells

Pancreatic acinar cells were prepared by collagenase digestion, as described previously (Wen et al., 2018). Isolated pancreatic acinar cells were incubated at 37°C in DMEM/F-12 medium containing 10% fetal bovine serum with or without CCK or BRD4 inhibitor (JQ1) or chloroquine (CQ) (Sigma, #C6628) or SIRT1 inhibitor (EX527). For viral transduction, cells were infected with 10^7^ plaque forming unit per ml adenovirus 24 h before stimulation. The siRNA sequence used for viral transduction is CCATGGATATGGGAACAAT (#1), GCCTCCAAAGAAGGATGTA (#2), GCCTGAAGAGCCAGTTGTT (#3), and TTCTCCGAACGTGTCACGT (Negative Control).

### ATP Measurement

ATP levels in acinar cells were detected by using the Cell Titer Glo Luminescent Cell Viability Assay kit (Promega, Madison, WI) according to the manufacturer’s instructions, as previously described (Han et al., 2017). In brief, cells (3.0 × 10^6^/ml) were treated with JQ1 (500 nmol/L) for 1 h, prior to CCK (200 nmol/L) treatment for 4 h. After the treatment, add 100 ul cell suspension into 96-well culture plate. Then add the ATP depletion reagents, and detect the level of bioluminescence using a Synergy multifunctional Microplate Reader (Gene Company Ltd, China). Data were normalized to protein concentration for each sample, then normalized to the untreated controls as 100%.

### Assessment of PI Uptake

Isolated pancreatic acinar cells (3.0 × 10^6^ per ml) were treated with JQ1 (500 nmol/L) for 1 h, prior to CCK (200 nmol/L) treatment for 4 h. Then cells were treated with propidium iodide (PI; 1 μmol/ml) for 5 min and the fluorescent intensity (excitation 536, emission 617), as PI uptake by the cells, was detected using a Synergy multifunctional Microplate Reader. Then 10 µl of 25% Triton-X100 (Sigma, #T8787) was added into the cells, and shake for 10 min and the fluorescent intensity (excitation 536, emission 617) was measured, as total amount of the cells. The percentage of PI uptake was calculated by Read 1 dividing Read 2 (% PI uptake = Read 1/Read 2 ×100).

### Measurement of LDH Release

Detecting necrosis in pancreatic acinar cells was used the method of determining LDH released into the cultured medium, as reported earlier (Gukovskaya et al., 1997; Mareninova et al., 2006; Sung et al., 2009). In brief, cells (3.0 × 10^6^/ml) were treated with JQ1 (500 nmol/L) for 1 h, prior to CCK (200 nmol/L) treatment for 4 h. LDH release was measured using LDH Cytotoxicity Assay Kit (Beyotime, Shanghai, China. C0017) according to the manufacturer’s instructions. The absorbance at 490 nm was detected by using Microplate Reader (BioTek Instruments, USA).

### LysoTarcker Red Staining

Isolated pancreatic acinar cells (3.0 × 10^6^/ml) were treated with JQ1 (500 nmol/L) for 1 h, prior to CCK (200 nmol/L) treatment for 4 h and then were harvested. After incubating with 500 μl of pre-warmed medium containing 75 nmol/L LysoTracker Red DND-99 dye (excitation 577 nm, emission 590 nm; 40739ES50, Yeasen, China) for 1 h, cells were washed and resuspended with Hoechst 33528 (40730ES10, Yeasen, China) for 15 min at 37°C. Lysosomal function was imaged by confocal imaging (Leica, Wetzlar, Germany).

### Measurement the Activities of Cathepsin B and Cathepsin L

The activities of cathepsin B and cathepsin L were measured by the Cathepsin B Assay Kit (Abcam, #ab65300) and Cathepsin L Activity Assay Kit (Abcam, #ab65306), respectively, according to the manufacturer’s instructions. Briefly, isolated pancreatic acinar cells (3.0 × 10^6^/ml) were pre-treated with JQ1 (500 nmol/L) with or without 10 μmol/L EX527 for 1 h, prior to CCK (200 nmol/L) stimulation for 4 h. The cell lysate and reaction buffer were added to a 96‐well black plate (Block Plate, WHB, Shanghai, China). The fluorescent intensity (excitation 400, emission 505) for both was detected using a Synergy multifunctional Microplate Reader.

### Induction of Experimental Acute Pancreatitis

CER hyperstimulation pancreatitis was induced by ten hourly intraperitoneal injections of CER (100 μg/kg) (Lerch and Gorelick, 2013). Controls received similar injections of physiologic saline. JQ1 (20 mg/kg) was injected *via* tail vein 1 h before the first injection of CER. Mice were anesthetized by pentobarbital sodium (1.5%, w/v) 12 h after the ﬁrst injection of CER, then blood and pancreas were collected.

Fatty acid ethyl ester pancreatitis model was induced by two hourly intraperitoneal injections of POA (150 mg/kg) and ethanol (1.35 g/kg) (Huang et al., 2014; Wen et al., 2015). The control animals were injected with equal volumes of ethanol. JQ1 (20 mg/kg) was injected *via* tail vein 1 h before the first injection of POA and ethanol. Mice were anesthetized by pentobarbital sodium (1.5%, w/v) 24 h after the ﬁrst injection of POA and ethanol, then blood and pancreas were harvested.

L-Arg pancreatitis model was induced by two hourly intraperitoneal injections of 8% L-Arg (pH = 7.0), at a dose of 4 g/kg body weight (Dawra et al., 2007). Control group were injected with equal volume of saline. Mice were killed humanely by cervical dislocation 72 h after the first L-Arg injection and pancreas was harvested.

NaT pancreatitis was induced by pancreatic duct retrograde injection of 2% NaT (5 μl/min by infusion pump for 10 min), which is described previously (Perides et al., 2010). Control mice received the laparotomy only. After 24 h, mice were humanely killed by cervical dislocation pancreas were collected.

### Serum Amylase

Blood samples were collected and centrifuged for 10 min at 3,000 rpm in 4°C. Serum amylase levels were detected by enzyme dynamics chemistry using commercial kits according to the manufacturer’s protocols (Roche, Basel, Switzerland).

### Hematoxylin–Eosin Staining and Immunohistochemistry

H&E staining was performed after being fixed in 4% paraformaldehyde and embedded in paraffin, tissues were cut into 4 μm sections. Pancreatic sections were scored by two pathologists in a blind manner for edema, inflammatory infiltration, and necrosis, ranging from 0 to 3 (Wildi et al., 2007). CD45 antibody (1:100) was used for immunohistochemistry to evaluate pancreatic inflammatory cell infiltration (Wen et al., 2018). Briefly, after deparaffinization and antigen retrieval by proteinase K, non-specific bindings were blocked by 5% bovine serum albumin, and then primary antibody was incubated overnight at 4°C. Sections were treated with alkaline phosphatase labeled secondary antibody for 1 h and then imaged by substrate tablets (Sigma-Aldrich, St. Louis, MO, USA).

### Immunofluorescence

The pancreas samples were embedded in paraffin and then deparaffinized. Antigen retrieval was performed by sodium citrate buffer (pH= 6), and then sections were blocked by 5% bovine serum albumin for 1 h. Sections were stained with monoclonal antibody against BRD4 (1:200) and amylase (1:300) overnight at 4°C; Alexa Fluor 488-labeled secondary antibody (1:200) and Alexa Fluor 488-labeled secondary antibody (1:300) for 1 h. Nuclei were stained with 4′,6-diamidino-2-phenylindole (DAPI) for 10 min. Sections were imaged by confocal microscope.

### Western Blot

Total protein of pancreatic tissue and pancreatic acinar cells were lysed with RIPA which contained protease and phosphatase inhibitors, as previously described (Han et al., 2017). Proteins were loaded on a 15% or 10% polyacrylamide gel. Primary antibodies LC3B (1:1,000), p62 (1:1,000), AMPK (1:1,000), phosphorylated AMPK (1:1,000), mTOR (1:1,000), phosphorylated mTOR (1:1,000), ATG14 (1:1,000), STX17 (1:1,000), cathepsin B (1:1,000), cathepsin L (1:1,000), LAMP2 (1:1,000), BRD4 (1:800), and β-actin (1:1000) were used. The protein bands were detected by chemiluminescence (Millipore, USA) using Amersham Imager 600 (GE Healthcare, USA) and quantified by using Image J software.

### Quantitative Reverse Transcription PCR (qRT-PCR)

Total RNA from the pancreatic acinar cells or pancreatic tissues was isolated using Trizol reagent (Takara, Japan). Reverse transcription was used PrimeScript RT Master Mix (Perfect Real Time) (Takara, Japan). Gene expression was detected by real-time PCR using TB Green chemistry (Takara, Japan) on a QuantStudio 6 Flex System using gene-speciﬁc, intron-spanning primers (**Table 1**). The results were normalized to β-actin and expressed as fold changes over control group.

**Table 1:** Primer sequences used for qRT-PCR.

Gene name	Forward primers (5′ →3′)	Reverse primers(5′ →3′)
*Brd4*	AAATCAGCTCACCAGGCTGT	TCTTGGGCTTGTTAGGGTTG
*Tnf*	TCTCTTCAAGGGACAAGGCTG	ATAGCAAATCGGCTGACGGT
*Il1b*	TTGACGGACCCCAAAAGAT	GAAGCTGGATGCTCTCATCTG
*Il6*	TTCATTCTCTTTGCTCTTGAATTAGA	GTCTGACCTTTAGCTTCAAATCCT
*Map1lc3b*	CGTCCGAGAAGACCTTCAAGCAG	TGCGGCAGGAGAACCTACTGG
*Vmp1*	*TAAGGATCAGCACAATGGAAGT*	TCCAGAGAGAAATACTGCAAGG
*Atg2a*	GCTGCTCAGTGCCGTCAACC	AGAAGAAGAGGTCCGTGCTGTCC
*Becn1*	GGCCAATAAGATGGGTCTGA	GCTGCACACAGTCCAGAAAA
*Sirt1*	ATCGGCTACCGAGACAAC	GTCACTAGAGCTGGCGTGT
*β-actin*	ATGGAGGGGAATACAGCCC	TTCTTTGCAGCTCCTTCGTT

### Statistical Analysis

Data were showed as mean ± SEM and analyzed by GraphPad Prism 4.0c (GraphPad Software, Inc.). The comparison between two groups was determined by Student’s un-paired, two-tailed t-test. P value <0.05 was considered statistically signiﬁcant.

## Results

### BRD4 Is Upregulated in Mouse Models of Acute Pancreatitis

**Figure 1:**
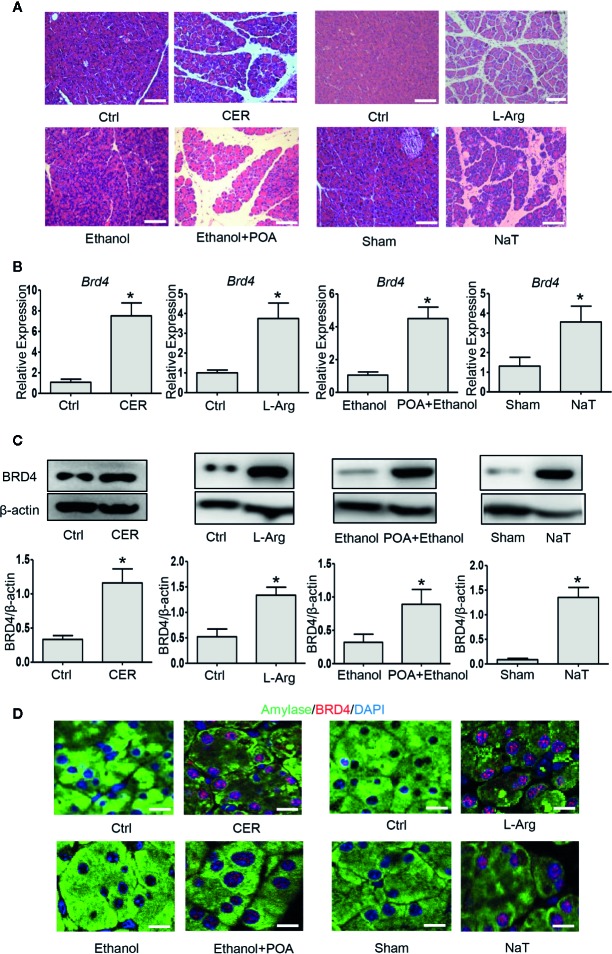
BRD4 is upregulated during various models of experimental acute pancreatitis. **(A)** Representative H&E images of pancreatic sections in CER, L-Arg, Ethanol- and POA, and NaT (200×), scale bar: 100 μm. **(B)** mRNA levels of *Brd4*. **(C)** Immunoblot analysis of BRD4 from pancreatic tissue. **(D)** Double immunofluorescent staining of BRD4 (red) and amylase (green) from pancreatic tissues (400×). 4′,6-Diamidino-2-phenylindole (DAPI; blue) was used to counterstain nuclei. Scale bar: 20 μm. Data represent the mean values ± SEM (n = 5). Statistical analysis was performed by Student’s un-paired, two-tailed t-test between two groups, *P < 0.05 versus control.

To examine the role of BRD4 during experimental AP, we firstly evaluated the expression of BRD4 in various clinically representative mouse models of AP. We found that the mRNA and protein expression of BRD4 were markedly upregulated in caerulein hyperstimulation pancreatitis, L-arginine-induced pancreatitis representing severe form of AP (Dawra et al., 2007), fatty acid ethyl ester pancreatitis mimicking alcohol associated acute pancreatitis (Huang et al., 2014), and NaT pancreatitis mimicking biliary acute pancreatitis (Perides et al., 2010) (**Figures 1A–C**). Consistently, immunofluorescent co-staining for BRD4 and amylase revealed that there was virtually undetectable expression of BRD4 in the exocrine of the pancreas in normal mice (**Figure 1D**). In contrast, BRD4 expression was markedly increased in the pancreas during four models of AP (**Figure 1D**). These results suggest that BRD4 may play a role in the pathogenesis of AP.

### BRD4 Inhibition Protects Against Pancreatic Acinar Cell Injury

**Figure 2:**
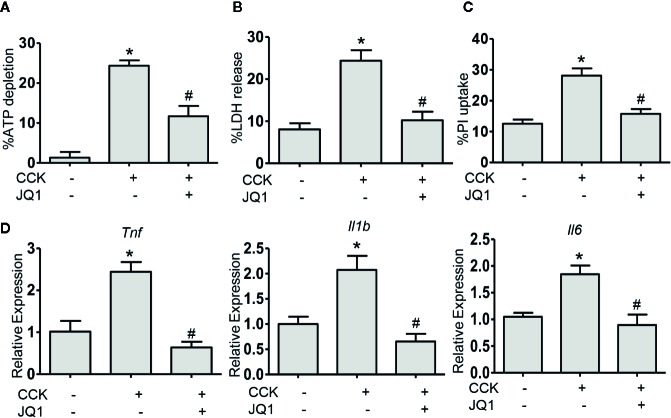
BRD4 inhibition prevents the activation of cell death pathway and downregulation of proinflammatory gene expression *in vitro*. **(A)** ATP levels were measured by luminescence in isolated pancreatic acinar cells. Data were normalized to untreated control as 100% (n = 5). **(B)** The percentage of total cellular lactate dehydrogenase (LDH) released into the extracellular medium in isolated pancreatic acinar cells stimulated with CCK (200 nmol·L^-1^) in the absence or presence of JQ1 (500 nmol·L^-1^) (n = 5). **(C)** PI uptake induced by CCK (200 nmol·L^-1^) with or without JQ1 (500 nmol·L^-1^) in isolated pancreatic acinar cells (n = 5). **(D)** mRNA levels of *Tnf*, *Il1b*, and *Il6* in CCK-stimulated pancreatic acinar cells with or without JQ1 (n = 3). Data represent the mean values ± SEM. Statistical analysis was performed by Student’s un-paired, two-tailed t-test between two groups, *P < 0.05, compared to the control; ^#^P < 0.05, compared to CCK-stimulated group.

In order to evaluate the therapeutic benefit of BRD4 in AP, we measured the effect of BRD4 inhibition on CCK-induced pancreatic acinar cell injury *in vitro.* In isolated primary pancreatic acinar cells, stimulation with CCK resulted in a marked reduction in the intracellular ATP levels and treatment with BRD4 inhibitor (JQ1) prevented CCK-induced loss of intracellular ATP (**Figure 2A**). To further confirm the effect of BRD4 inhibition on pancreatic acinar cell necrosis, we quantified acinar cell necrosis by LDH release and PI uptake. Inhibition of BRD4 by JQ1 markedly inhibited CCK-induced LDH release and the percentage of PI uptake, suggesting that BRD4 inhibition protected against necrotic cell death pathway activation in primary pancreatic acinar cells (**Figures 2B, C**). Moreover, we found that BRD4 inhibition also evidently inhibited the expression of several pro-inflammatory mediators, including *Tnf*, *Il1b*, and *Il6* (**Figure 2D**). These results demonstrate that BRD4 inhibition protects against CCK-induced pancreatic acinar cell injury and inflammation.

### BRD4 Inhibition Restores Impaired Autophagic Flux *In Vitro* and the Effect of BRD4 Inhibition Is Mediated by Upregulating SIRT1

**Figure 3:**
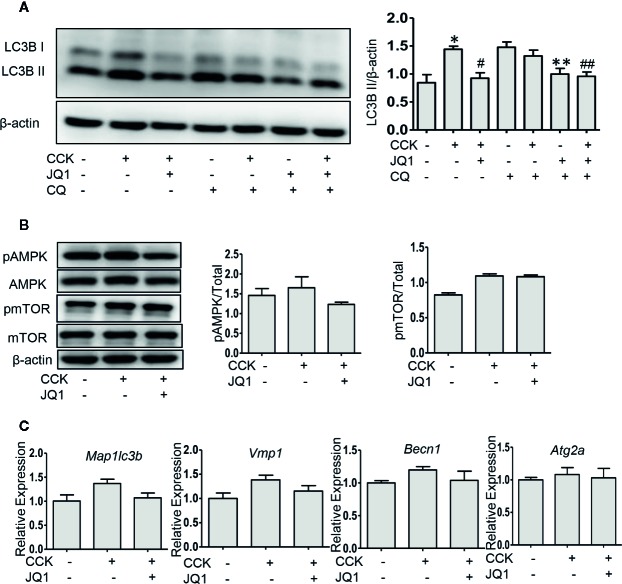
BRD4 inhibition restores impaired autophagic influx, but has no effect on autophagy initiation. **(A)** Immunoblot analysis of LC3B level at 4 h after CCK or CQ stimulation in isolated pancreatic acinar cells (n = 3). **(B)** Immunoblot analysis of AMPK, mTOR phosphorylation levels at 1 h after CCK stimulation in isolated pancreatic acinar cells (n = 3). **(C)** mRNA levels of *Map1lc3b*, *Vmp1*, *Becn1*, and *Atg2a* at 4 h after CCK stimulation (n = 3). Data represent the mean values ± SEM. Statistical analysis was performed by Student’s un-paired, two-tailed t-test between two groups, *P < 0.05, compared to the control; #P < 0.05, compared to CCK-stimulated group;^##^P < 0.05, compared to the CQ-stimulated group; ^#^P < 0.05, compared to CCK+CQ-stimulated group.

**Figure 4:**
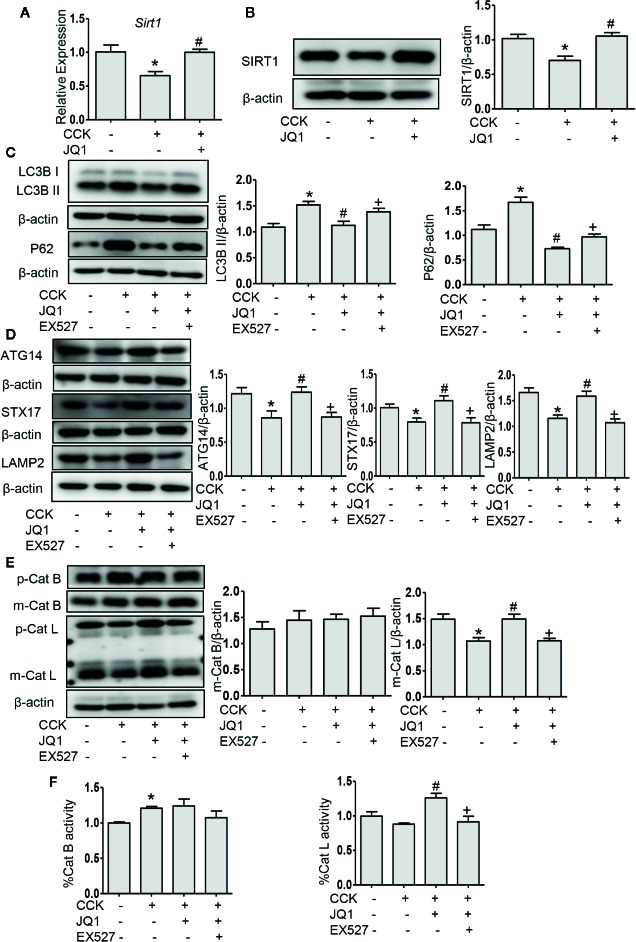
BRD4 inhibition upregulated SIRT1 and inhibition of SIRT1 reversed the effects of BRD4 inhibition on autophagic flux. **(A)** mRNA level of *Sirt1* at 4 h after CCK stimulation in isolated pancreatic acinar cells (n = 3). **(B)** Immunoblot analysis of SIRT1 level at 4 h after CCK stimulation (n = 5). **(C)** Immunoblot analysis for LC3B and p62 expression in isolated pancreatic acinar cells pretreated with 500 nmol·L^-1^ JQ1 or 10 μmol·L^-1^ EX527 followed by stimulation with 200 nmol·L^-1^ CCK for 4 h (n = 4). **(D)** Immunoblot analysis for ATG14, STX17, and LAMP2 expression (n = 5). **(E)** Immunoblot analysis for cathepsin B (n = 3) and cathepsin L expression (n = 5). **(F)** The activities of cathepsin B (n = 3) and cathepsin L (n = 3). Data represent the mean values ± SEM. Statistical analysis was performed by Student’s un-paired, two-tailed t-test between two groups, *P < 0.05, compared to the control; ^#^P < 0.05, compared to CCK-stimulated group; ^+^P < 0.05, compared to JQ1-treated group.

Impaired autophagy plays an important role in the pathogenesis of AP (Piplani et al., 2019). It has been shown that a series of autophagy-associated genes, including induction of autophagy, the fusion of autophagosome with lysosome and lysosomal degradation are repressed by BRD4 (Sakamaki et al., 2017), we next sought to determine whether BRD4 influences autophagic flux in AP. Firstly, we found that the protein expression of the LC3B-II and p62 was significantly elevated in pancreatic acinar cells after CCK stimulation. Treatment with chloroquine (CQ) in the presence or absence of CCK, an well-known inhibitor of autophagy that blocks lysosomal degradation, similarly led to an increase in the protein expression of the LC3B-II. Compared to CCK-stimulated and CQ-treated cells, inhibition or knockdown of BRD4 decreased the accumulation of LC3B-II and more markedly decreased the accumulation of p62, indicating that BRD4 inhibition may enhance autophagic flux (**Figure 3A**; **Figures S1A, B**). Next, we assessed the effect of BRD4 inhibition on AMPK and mTOR, which regulate autophagy induction through Ulk1 phosphorylation (Kim et al., 2011). Interestingly, we found that BRD4 inhibition had no significant effect on the activation of AMPK and mTOR, suggesting that inhibition of BRD4 did not affect the induction of autophagy in isolated pancreatic acinar cells (**Figure 3B**). We also found BRD4 inhibition did not affect the expression of the genes that were related to autophagosomes formation, including *Becn1*, *Vmp1*, *Atg2a*, and *Map1lc3b* (**Figure 3C**). Since the histone deacetylase, SIRT1 has been reported to regulate autophagy-lysosomal pathway (Hariharan et al., 2010; Huang et al., 2019) and can be upregulated by BRD4 inhibition (Kokkola et al., 2015). We examined whether BRD4 inhibition restores impaired autophagy by upregulating SIRT1. As expected, the expression of SIRT1 was upregulated by JQ1 treatment or BRD4 knockdown in isolated pancreatic acinar cells (**Figures 4A, B**; **Figure S4**). Furthermore, compared with JQ1-treated group, treatment with the SIRT1 inhibitor EX527 resulted in an increased expression of the autophagic markers such as LC3B-II and p62 (**Figure 4C**).

Autophagosome fusion with lysosome is a key step in autophagy progression, which is regulated by ATG14 and the complex formed by STX17, synaptosome associated protein 29, and vesicle associated membrane protein 8 (Itakura and Mizushima, 2013). We measured the expression of ATG14, STX17, and LAMP2 and found that the protein levels of ATG14, STX17, and LAMP2 were downregulated in pancreatic acinar cells after CCK stimulation and inhibition of BRD4 by JQ1 or knockdown of BRD4 by transfecting shRNA restored their expression (**Figure 4D**; **Figures S2A, B**), suggesting that BRD4 inhibition enhances the fusion of autophagosomes with lysosomes. Furthermore, using LysoTracker staining, we found that BRD4 inhibition maintained lysosomal pH (**Figure S3**). Interestingly, we also observed that treatment with the SITR1 inhibitor, EX527 reversed the effects of BRD4 inhibition on the expression of autophagosome and lysosome fusion markers, including ATG14, STX17, and LAMP2 (**Figure 4D**), indicating the influence of BRD4 inhibition on autophagosome fusion with lysosome was mediated by SIRT1.

It has been shown that during AP, impaired autophagy is related to an imbalance between cathepsin L and cathepsin B as the former degrades trypsinogen and trypsin into amino acid and the latter converts trypsinogen into trypsin (Mareninova et al., 2009). We next measured the expression of cathepsin L and cathepsin B and found that inhibition or knockdown BRD4 significantly increased the level of the pro-enzyme and cleaved form of cathepsin L. Interestingly, BRD4 inhibition did not alter the expression of cathepsin B, but BRD4 knockdown markedly decreased the expression of cathepsin B (**Figure 4E**; **Figure S2C**), suggesting that BRD4 mediates the balance between cathepsin L and cathepsin B, therefore, resulting in reduced pancreatic acinar cell injury. And with the SITR1 inhibitor treatment, the pro-enzyme and the cleaved form of cathepsin L was decreased, but the level of cathepsin B was not changed (**Figure 4E**). To further assess the effect of JQ1 on lysosomal function, we measured the activity of cathepsin B and L. BRD4 inhibition increased the activity of cathepsin L and SIRT1 inhibitor reversed its effect, but there was no effect on the activity of cathepsin B (**Figure 4F**). Taken together, these data suggest that BRD4 inhibition enhances the fusion of autophagosome with lysosome and improves lysosomal degradation, but has no influence on autophagy initiation and autophagosome formation. Secondly, the influence of BRD4 inhibition on autophagy was likely mediated by upregulating SIRT1.

### BRD4 Inhibition Alleviates Two Acute Pancreatitis Models

**Figure 5:**
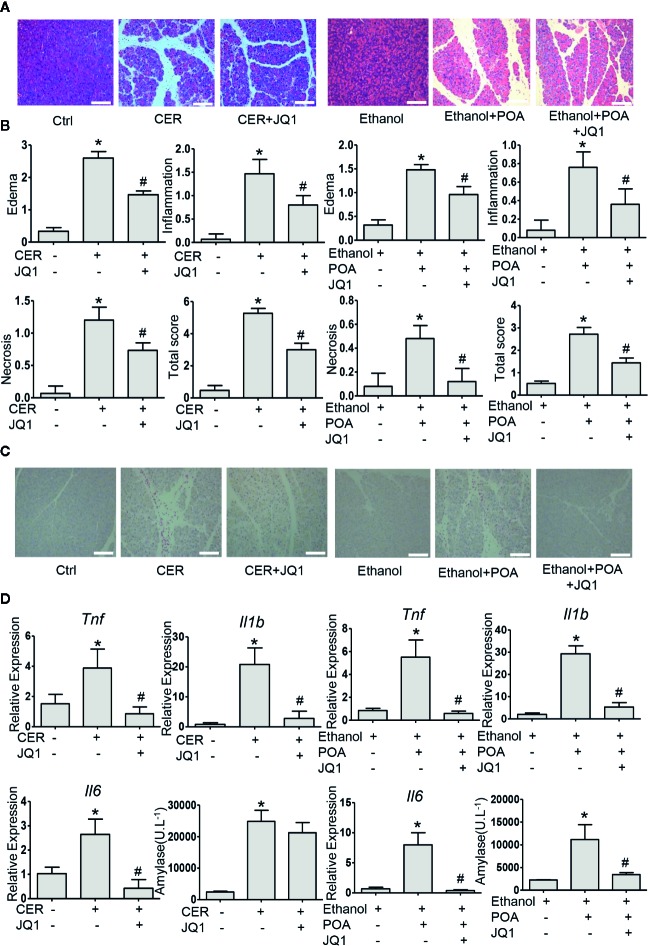
BRD4 inhibition protects against caerulein- and ethanol plus POA-induced pancreatitis *in vivo.* **(A)** Representative H&E images of pancreatic sections in CER and ethanol plus POA-induced pancreatitis (200×) (n = 5). **(B)** Histopathological scores for edema, inflammation, necrosis, and the total histology score (n = 5). **(C)** Representative micrographs of leukocyte marker CD45 immunohistochemical staining for the pancreas (200×). **(D)** mRNA levels of *Tnf*, *Il1b*, and *Il6* from pancreatic tissue (n = 3) and serum amylase levels (n = 5). Scale bar = 100 μm. Data represent the mean values ± SEM. Statistical analysis was performed by Student’s un-paired, two-tailed t-test between two groups, *P < 0.05, compared to the control; #P < 0.05, compared to AP group.

Then, we examined the influence of BRD4 inhibition on pancreatitis severity during two experimental models of AP *in vivo*. Firstly, we tested JQ1, the inhibitor of BRD4 in caerulein hyperstimulation pancreatitis, a widely used and highly reproducible AP model (Lerch and Gorelick, 2013). We showed that JQ1 markedly reduced pancreatic edema, inflammatory infiltration, and necrosis (**Figures 5A, B**). Furthermore, leukocyte infiltration assessed by CD45 immunostaining and pro-inflammatory cytokines such as *Tnf*, *Il1b*, and *Il6* in the pancreas were decreased with JQ1 treatment (**Figures 5C, D**). The second AP model was induced by administrations of POA and ethanol, which is associated with alcohol-induced pancreatitis (Huang et al., 2014). Similarly, we found that JQ1 significantly reduced histological scores in the pancreas, including edema, inflammation, and necrosis (**Figures 5A, B**). Furthermore, JQ1 decreased leukocyte infiltration and inflammatory factors in the pancreas and serum amylase levels in this model (**Figures 5C, D**). Consistently with our *in vitro* findings, these data showed that BRD4 inhibition protects against two clinically relevant models of AP.

### BRD4 Inhibition Restores Impaired Autophagy *In Vivo*

**Figure 6:**
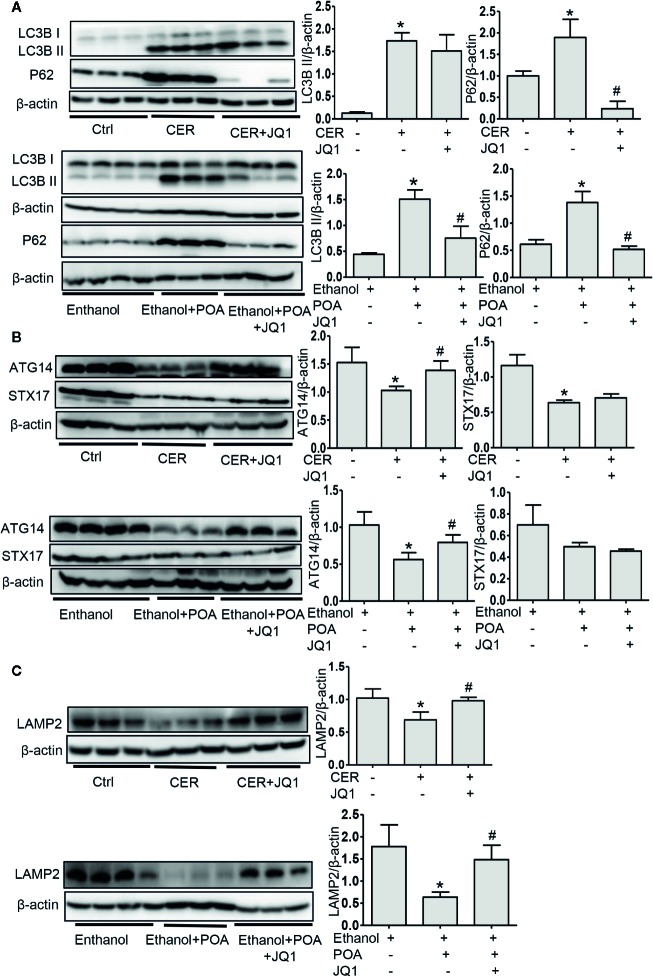
BRD4 inhibition restores impaired autophagic influx *in vivo via* promoting autophagosome-lysosome fusion and lysosomal degradation. **(A)** Immunoblot analysis for LC3 and p62 in the pancreas from CER (upper) or ethanol plus POA (lower). **(B)** Immunoblot analysis for ATG14 and STX17 in the pancreas from CER (upper) or ethanol plus POA (lower). **(C)** Immunoblot analysis for LAMP2 in the pancreas from CER (upper) or ethanol plus POA (lower). Data represent the mean values ± SEM (n = 3). Statistical analysis was performed by Student’s un-paired, two-tailed t-test between two groups, *P < 0.05, compared to the control; ^#^P < 0.05, compared to AP group.

Finally, we evaluated the effect of BRD4 inhibition on autophagy *in vivo*. In these two AP models, JQ1 treatment significantly downregulated p62 levels (**Figure 6A**). BRD4 inhibition downregulated LC3B-II expression in fatty acid ethyl ester-induced pancreatitis, while had no effect on LC3B-II expression CER model (**Figure 6A**). These data suggest that BRD4 inhibition restores impaired autophagy *in vivo*. Furthermore, we found that BRD4 inhibition upregulated the expression of ATG14 and LAMP2, but had no effect on STX17 in both models (**Figures 6B, C**), indicating BRD4 inhibition enhances the fusion of the autophagosome with lysosome *in vivo*. Finally, we detected the expression of SIRT1 *in vivo* and found that BRD4 inhibition significantly upregulated SIRT1 levels (**Figure S5**). Collectively, our data suggest that BRD4 inhibition *via* upregulating SIRT1 restores impaired autophagy *in vivo*.

## Discussion

The BET protein family includes four subtypes: Brd2, Brd3, Brd4, and Brd6/t which is testis specific. (Shi and Vakoc, 2014; Sakamaki et al., 2017). It has been known that among these isoforms only BRD4 knockdown mediates autophagy and lysosomal function (Sakamaki et al., 2017). Therefore, we focused our study on the role of BRD4 in AP. Among BRD4 inhibitors, we choose JQ1 since it is widely used pharmacological BRD4 inhibitor in various other diseases (Jiang et al., 2017; Rudman et al., 2018; Song et al., 2019). We found that BRD4 inhibition reduced CCK-induced pancreatic acinar cell injury and pro-inflammatory expression *in vitro*, and protected against two AP models. Interestingly, Huang et al. reported that treatment with I-BET-762, another BET inhibitor, markedly alleviated taurolithocholic acid-induced pancreatitis and POA plus ethanol AP, but not caerulein hyperstimulation pancreatitis (Huang et al., 2017), suggesting that different BET inhibitor may exert their anti-inflammatory effects through different mechanisms. As shown in our study, BRD4 inhibition by JQ1 protects against AP mainly through restoring impaired autophagic flux, which has been reported to be pivotal in the pathogenesis of AP.

Autophagy is a critical catabolic process which can degrade and recycle damaged organelles, lipids, and proteins (Habtezion et al., 2019). SQSTM1, also known as p62, is an autophagy receptor. As degraded along with cargos, it can be used as a marker of autophagic degradation (Klionsky et al., 2016). The excessive accumulation of p62 is a marker of impaired autophagy (Gukovskaya et al., 2017). We found that BRD4 inhibition and knockdown had a pronounced effect on reducing the accumulation of p62, indicating that BRD4 inhibition enhances autophagic degradation. It has been reported that BRD4 inhibition and knockdown promotes autophagy induction in early stages, the formation of autophagosomes, and subsequent fusion with lysosomes (Sakamaki et al., 2017). In our study, we found that BRD4 inhibition had no effects on the phosphorylation of mTOR and AMPK, two markers of autophagic induction, nor on the genes that were related to autophagosomes formation, suggesting that BRD4 inhibition did not affect autophagy induction and autophagosome formation. This may partly be explained by that pancreatic basal autophagy is high and is crucial for pancreatic hemostasis (Antonucci et al., 2015), but during experimental AP, activated autophagy is protective while incomplete autophagic process might be detrimental (Gukovskaya et al., 2017). Accumulating evidence demonstrated that autophagy in acute pancreatitis models is impaired (Mareninova et al., 2009; Gukovskaya et al., 2017; Biczo et al., 2018). The fusion of autophagosome with lysosome is critical for efficient lysosomal degradation and is regulated by a series of proteins, such as ATG14, Rab7, LAMP2, and STX17 (Itakura and Mizushima, 2013; Zhang et al., 2019). We found that BRD4 inhibition or knockdown increased the expression of ATG14, STX17, and LAMP2 in isolated pancreatic acinar cells and in two clinically relevant models of AP, suggesting that BRD4 inhibition restores impaired autophagy by improving the fusion of autophagosome with lysosome *in vitro* and *in vivo*.

In addition to the fusion of autophagosome with lysosome, efficient lysosomal degradation depends on the integrity of lysosome membrane and the activities of acidic hydrolases (Saftig and Klumperman, 2009). Cathepsin B and cathepsin L are involved in this process, cathepsin B converts trypsinogen to trypsin while cathepsin L degrades both trypsin and trypsinogen into amino acids (Halangk et al., 2000; Wartmann et al., 2010; Gukovskaya and Gukovsky, 2012). Previous studies have shown that in pancreatitis level of fully processed (mature) forms of these cathepsins decreased and accumulation of intermediate forms increased (Mareninova et al., 2009; Gukovskaya and Gukovsky, 2012; Biczo et al., 2018). Moreover, acidic environment in the lysosome plays a critical role in maintaining the normal activities of these enzymes (Mindell, 2012). In this study, we found that BRD4 inhibition maintained lysosomal pH, increased the expression of mature form of cathepsin L and the activity of cathepsin L in isolated pancreatic acinar cells, suggesting that BRD4 inhibition restores impaired autophagy during AP also by enhancing lysosomal degradation.

SIRT1 is a member of class III histone deacetylase, which is involved in the regulation of cell metabolism, apoptosis, and autophagy (Oellerich and Potente, 2012). It has been showed that BRD4 inhibitor JQ1 upregulated SIRT1 and alleviated inflammatory responses in a cellular model of lung disease (Kokkola et al., 2015). In addition, BRD4 inhibition induced ferritinophagy and downregulated the expression of genes that are related to ferroptosis by enhancing the expression of SIRT1 or suppressing the expression of the histone methyltransferase G9a (Sui et al., 2019). Consistently, we found that BRD4 inhibition or knockdown upregulated SIRT1 in pancreatic acinar cells and in experimental models of AP. It has been reported that SIRT1 regulates autophagy by interacting with autophagy related genes and deacetylating them. For example, SIRT1 deacetylate autophagy genes such as *Atg5*, *Atg7*, and *Atg8*, which is critical for the activation of autophagy induced by starvation (Lee et al., 2008). Moreover, SIRT1 deacetylated FOXO1 (forkhead box O1), enhancing autophagosome-lysosome fusion (Hariharan et al., 2010; Huang et al., 2019). SIRT1 also deacetylates FOXO3 (forkhead box O3), leading Bnip3‐mediated autophagy (Kume et al., 2010). In addition, SIRT1 deacetylates TFEB (transcription factor EB), enhancing the expression of autophagy/lysosome-associated genes (Bao et al., 2016). In this study, we observed that the inhibition of SIRT1 deacetylation with EX527 reversed the effects of BRD4 inhibition on autophagic flux. Specifically, inhibition of SIRT1 reversed the increased expression of LC3B-II and p62 with BRD4 inhibition, downregulated the increased expression of ATG14, STX17, and LAMP2 with BRD4 inhibition, and decreased the pro-enzyme and the cleaved form and the activity of cathepsin L, but the level and the activity of cathepsin B was not changed. These data suggest that SIRT1 is a crucial mediator that is responsible for BRD4-mediated autophagy during AP.

## Conclusion

**Figure 7:**
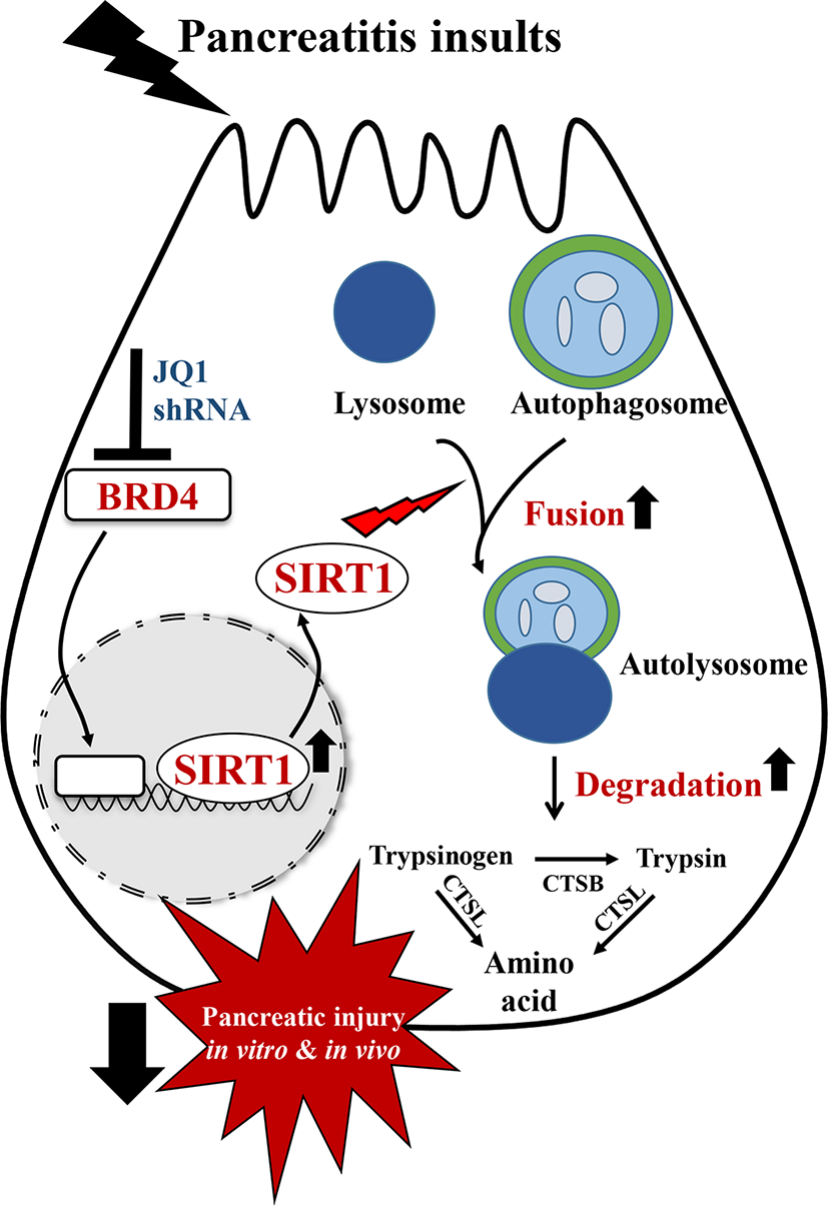
Schematic diagram for the mechanism of BRD4 inhibition on impaired autophagy during acute pancreatitis. BRD4 inhibition protects against pancreatic injury in the *in vitro* and *in vivo* settings of AP. Inhibition or knockdown of BRD4 upregulated SIRT1, leading to enhanced autophagosome fusion with lysosome and lysosomal degradation, therefore restored impaired autophagic flux by pancreatitis insults.

In summary, in this study, we showed that BRD4 expression is upregulated in various experimental models of pancreatitis. BRD4 inhibition alleviated pancreatic acinar cell injury and two clinically relevant mouse models of experimental AP. These protective effects were mediated by restoring impaired autophagic flux, primarily through enhancing autophagosome fusion with lysosome and lysosomal degradation. The regulation of impaired autophagic flux by BRD4 inhibition is through upregulating SIRT1 (**Figure 7**).

## Data Availability Statement 

All datasets generated for this study are included in the article/**Supplementary Material**.

## Ethics Statement 

All experiments involving animals were approved by the Institutional Animal Care and Research Ethics Committee of Shanghai Jiao Tong University School of Medicine (SYXK 2013–0050, Shanghai, China).

## Author Contributions

SS, BL, and JD performed the experiments and drafted the manuscript. SS, ZW, and YH performed the statistical analysis. LW, GH, and XW designed, conceived the study, revised the manuscript, and provided funding to support the study. All authors read and approved the submitted version.

## Funding

This work was sponsored by Natural Science Foundation of China to GH (81670584 and 81970556), XW (81570580), and LW (81900585), Shanghai Pujiang Program to GH (18PJD041) and LW (19PJ1408400), Shanghai Municipal Education Commission-Gaofeng Clinical Medicine Grant to LW.

## Conflict of Interest

The authors declare that the research was conducted in the absence of any commercial or financial relationships that could be construed as a potential conflicts of interest.

The reviewer OM declared a past co-authorship with one of the authors LW to the handling editor.

## Abbreviations

AP, acute pancreatitis; BRD4, Bromodomain-containing protein 4; BET, bromodomain and extraterminal; CCK, cholecystokinin; CQ, chloroquine; SIRT1, sirtuin 1; Atg, autophagy genes; CER, caerulein; POA, palmitoleic acid; L-Arg, L-Arginine; NaT, Na taurocholate; LAMP2, lysosomal-associated membrane protein 2; AMPK, AMP activated protein kinase; LC3B, microtubule associated protein 1 light chain 3 beta; STX17, syntaxin 17; mTOR, mammalian target of rapamycin; FOXO1, forkhead box O1; FOXO3, forkhead box O1; TFEB, transcription factor EB.
