# Supplementary material for: BRD4 Inhibition Protects Against Acute Pancreatitis Through Restoring Impaired Autophagic Flux
Source: Front Pharmacol. 2020 May 8;11:618. doi: 10.3389/fphar.2020.00618 (PMC7227015; doi:10.3389/fphar.2020.00618)
Supplement: Supplementary file 1 [file DataSheet_1.pdf]

Supplementary Material

1. Supplementary Figures

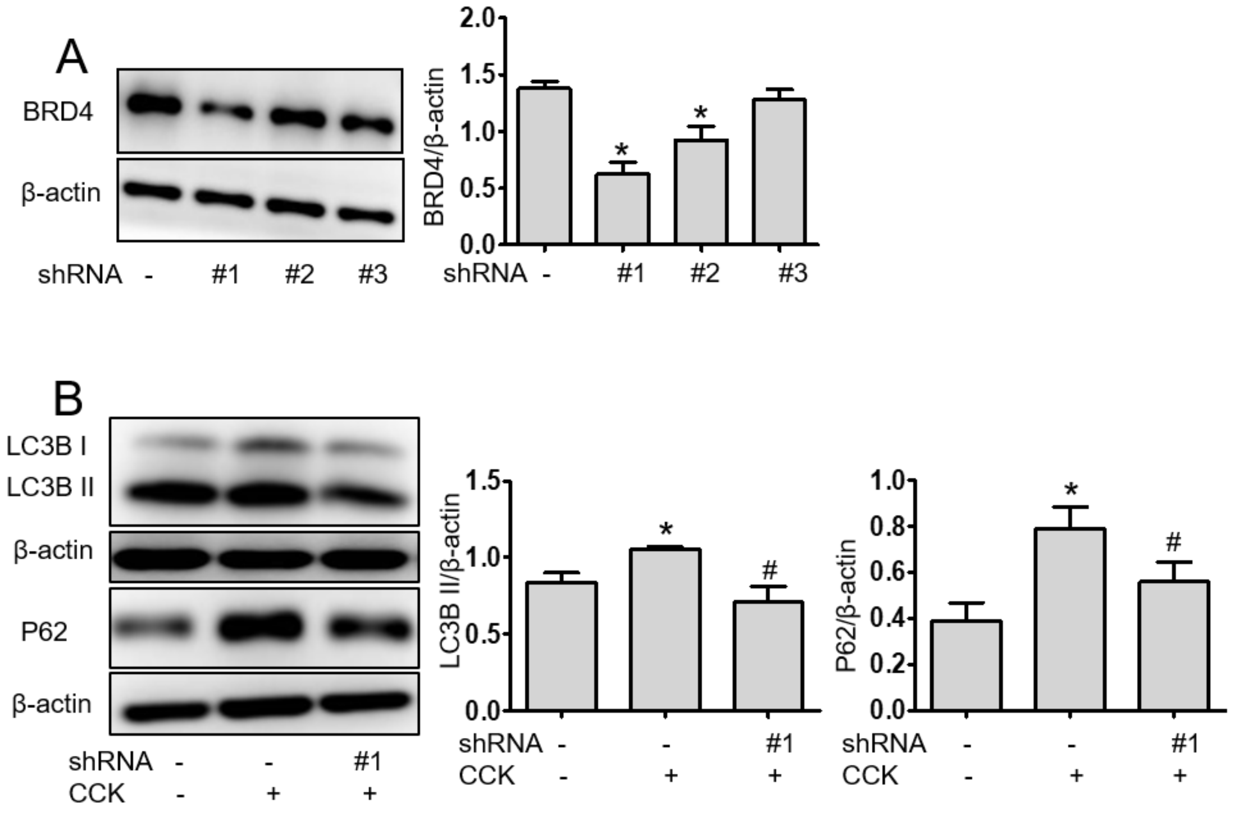

Supplementary Figure 1. BRD4 knockdown restores impaired autophagic flux. Primary pancreatic acinar cells were isolated from C57BL/6 mice and were infected by adenoviral with BRD4 or control shRNA. 24 h after infection, cells were stimulated with 200 nmol $\cdot L^{-1}$ CCK for an additional 4 h. **(A)** Immunoblot analysis of BRD4 expression to determine the knockdown efficiency of BRD4 shRNA. **(B)** Immunoblot analysis of LC3, p62 levels after BRD4 or control shRNA infection. Data were expressed as means ± SEM (n=3). Statistical analysis was performed by Student's un-paired, two-tailed t-test between two groups, *P < 0.05, compared to the control; #P < 0.05, compared to CCK-stimulated group.

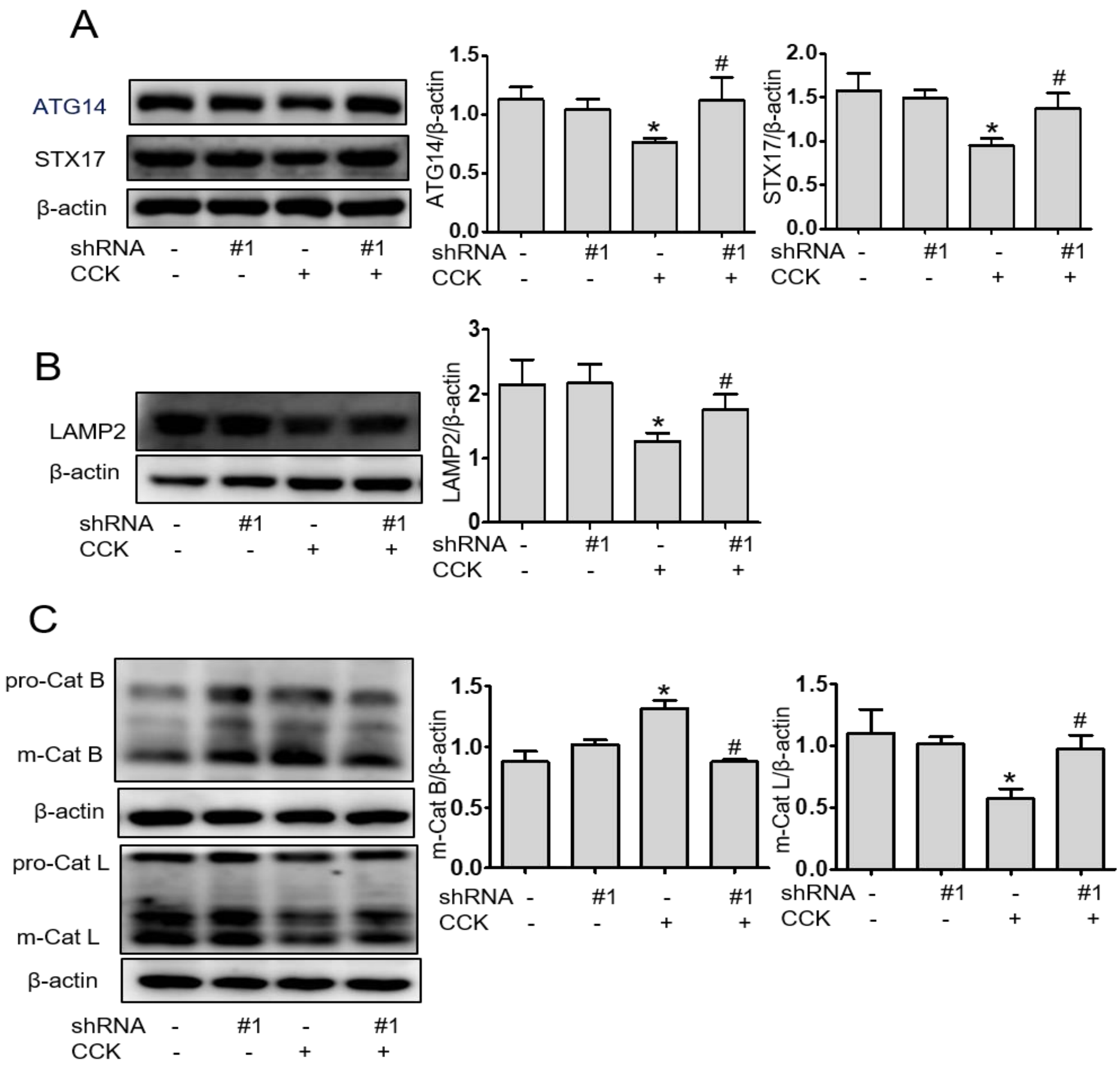

Supplementary Figure 2. BRD4 knockdown enhances autophagosome-lysosome fusion and lysosomal degradation. **(A)** Immunoblot analysis of ATG14, STX17 levels after BRD4 or control shRNA infection 24 h followed by CCK stimulation for 4 h. **(B)** Immunoblot analysis of LAMP2 levels after BRD4 or control shRNA infection 24 h followed by CCK stimulation for 4 h. **(C)** Immunoblot analysis of cathepsin B, cathepsin L levels. Data were expressed as means ± SEM (n =

3). Statistical analysis was performed by Student's un-paired, two-tailed t-test between two groups, *P < 0.05, compared to the control; #P < 0.05, compared to CCK-stimulated group.

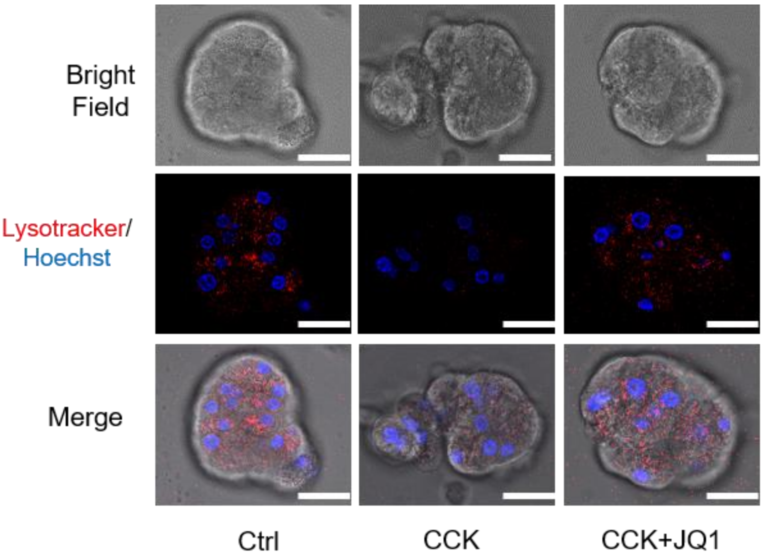

Supplementary Figure 3. BRD4 inhibition maintains lysosomal pH. Representative images depicting lysosomal acidic compartments by Lysotracker staining (630×). Scale bar: 50 μm.

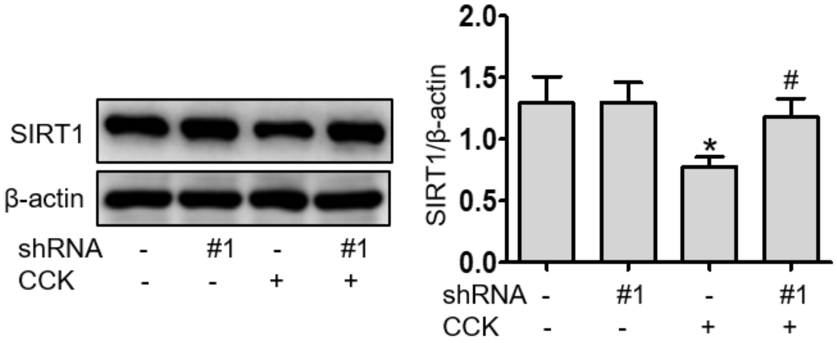

Supplementary Figure 4. BRD4 knockdown upregulated SIRT1 *in vitro*. Immunoblot analysis of SIRT1 expression after BRD4 or control shRNA infection 24 h followed by CCK stimulation for 4 h. Data were expressed as means ± SEM (n = 3). Statistical analysis was performed by Student's un-paired, two-tailed t-test between two groups, *P < 0.05, compared to the control; #P < 0.05, compared to CCK-stimulated group.

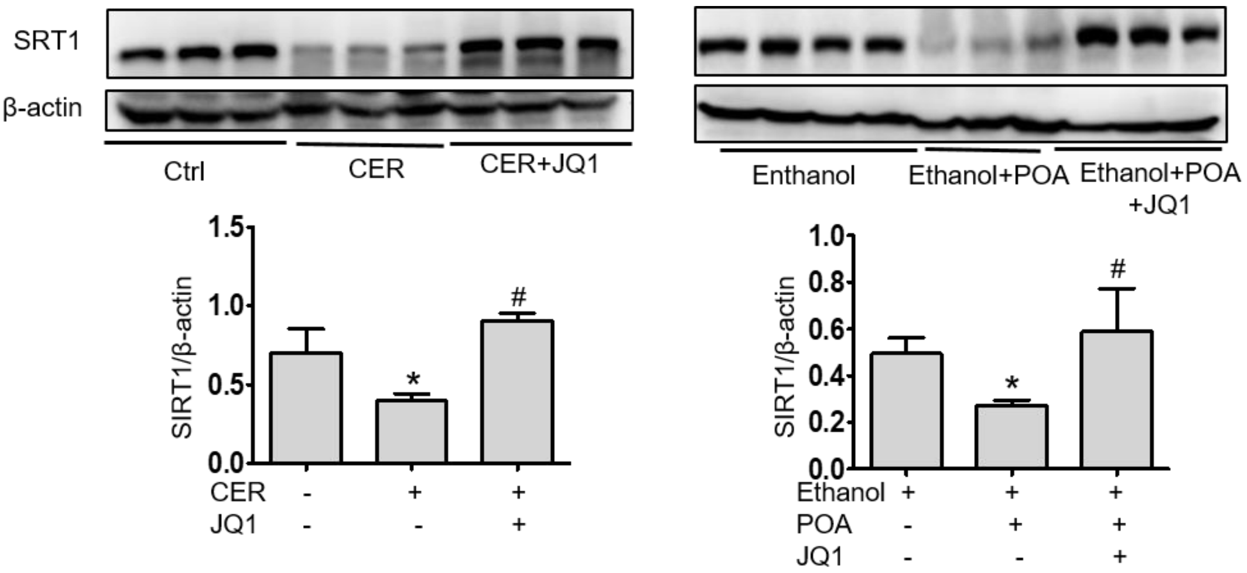

Supplementary Figure 5. BRD4 inhibition upregulated SIRT1 *in vivo*. Immunoblot analysis for SIRT1 in the pancreas from CER (left) or Ethanol + POA (right). Data were expressed as means ± SEM (n = 3). Statistical analysis was performed by Student's un-paired, two-tailed t-test between two groups, *P < 0.05, compared to the control; #P < 0.05, compared to AP.
