# Supplementary material for: BRD4 Inhibition Protects Against Acute Pancreatitis Through Restoring Impaired Autophagic Flux
Source: Front Pharmacol. 2020 May 8;11:618. doi: 10.3389/fphar.2020.00618 (PMC7227015; doi:10.3389/fphar.2020.00618)
Supplement: Supplementary file 2 [file DataSheet_2.pdf]

Figure 1

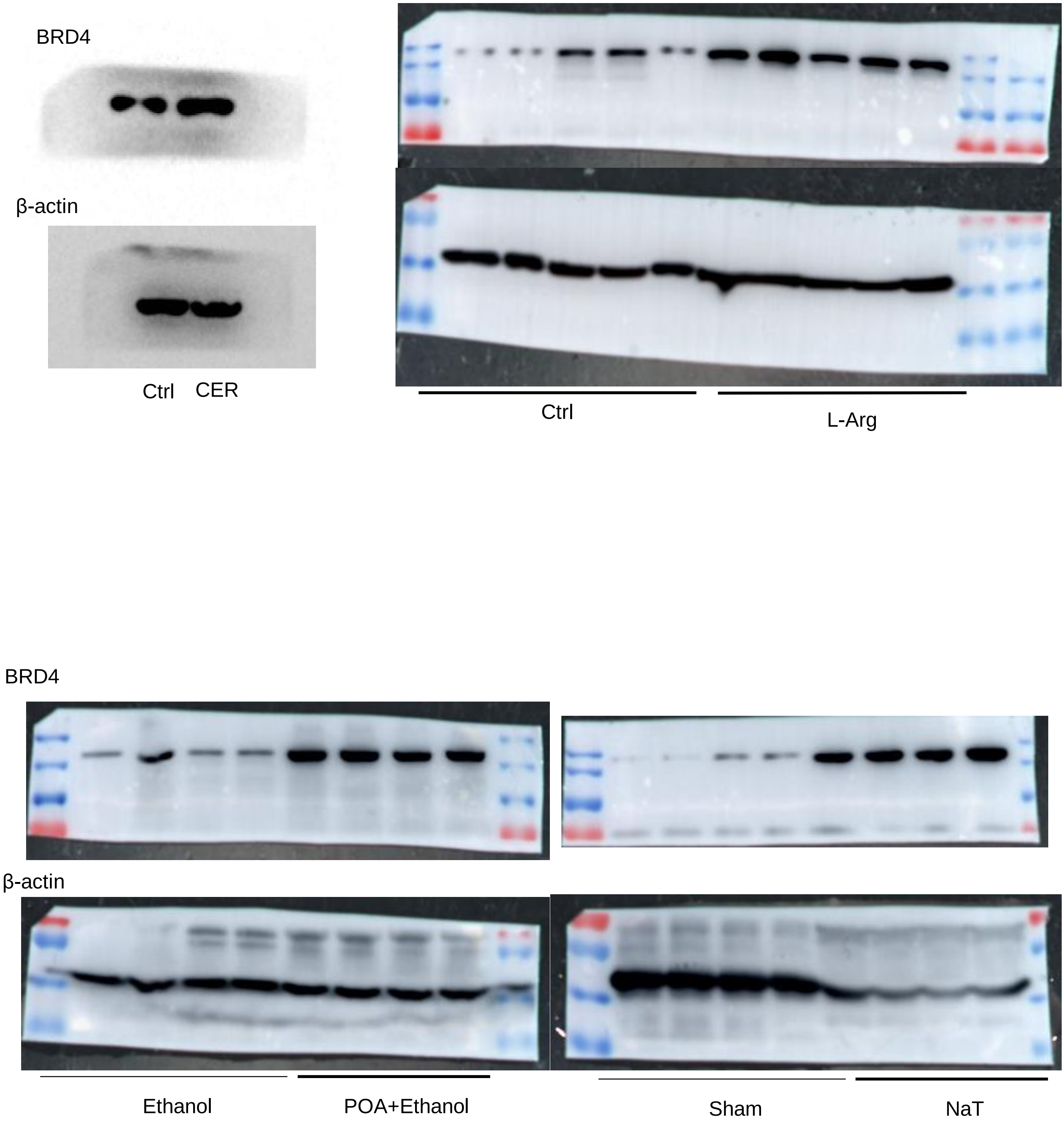
BRD4
β-actin
Ctrl
CER
Ctrl
L-Arg
BRD4
β-actin
Ethanol
POA+Ethanol
Sham
NaT

Figure 3

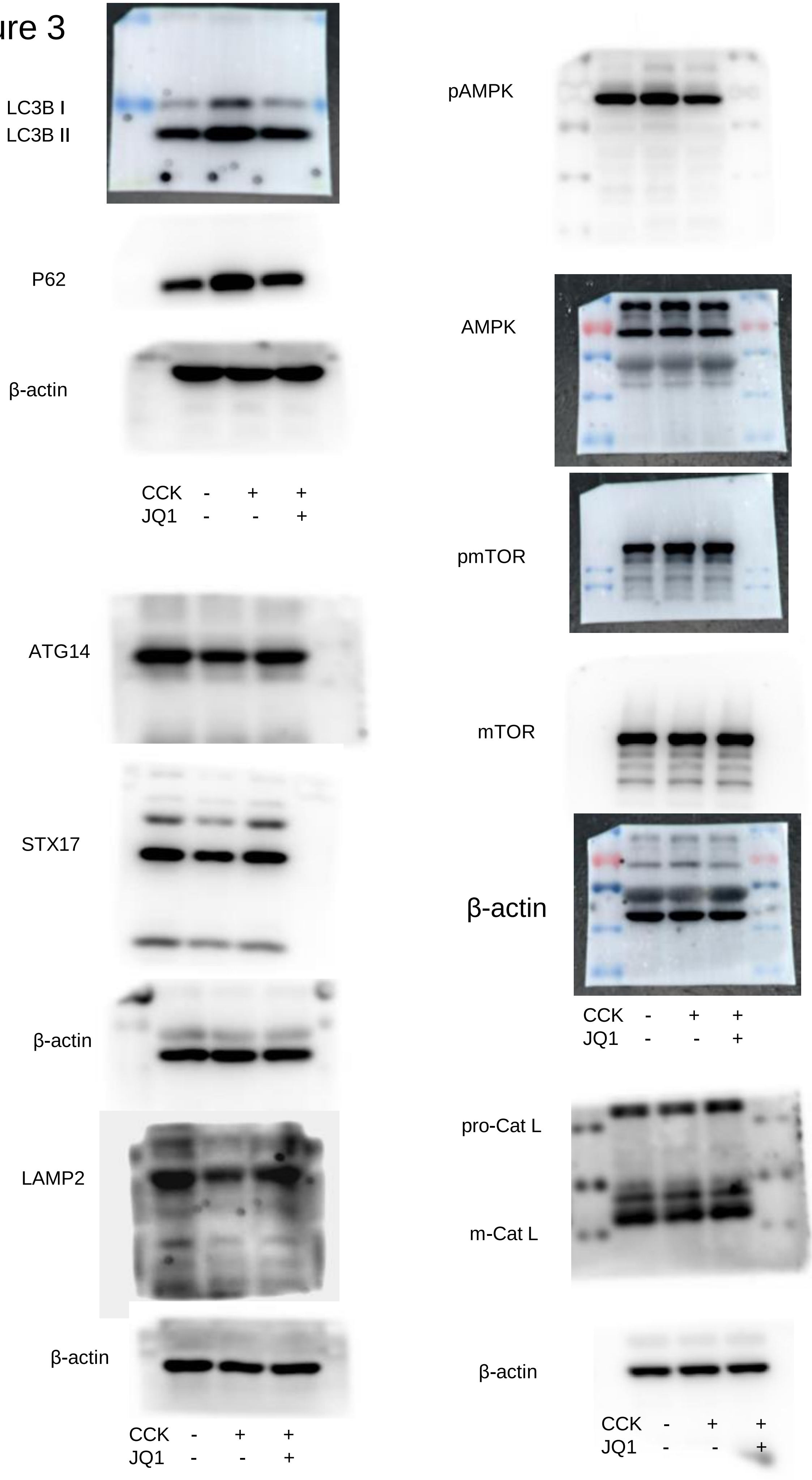
LC3B I
LC3B II
P62
β-actin
CCK - + +
JQ1 - - +
ATG14
STX17
β-actin
LAMP2
β-actin
CCK - + +
JQ1 - - +
pAMPK
AMPK
pmTOR
mTOR
β-actin
CCK - + +
JQ1 - - +
pro-Cat L
m-Cat L
β-actin
CCK - + +
JQ1 - - +

Figure 4

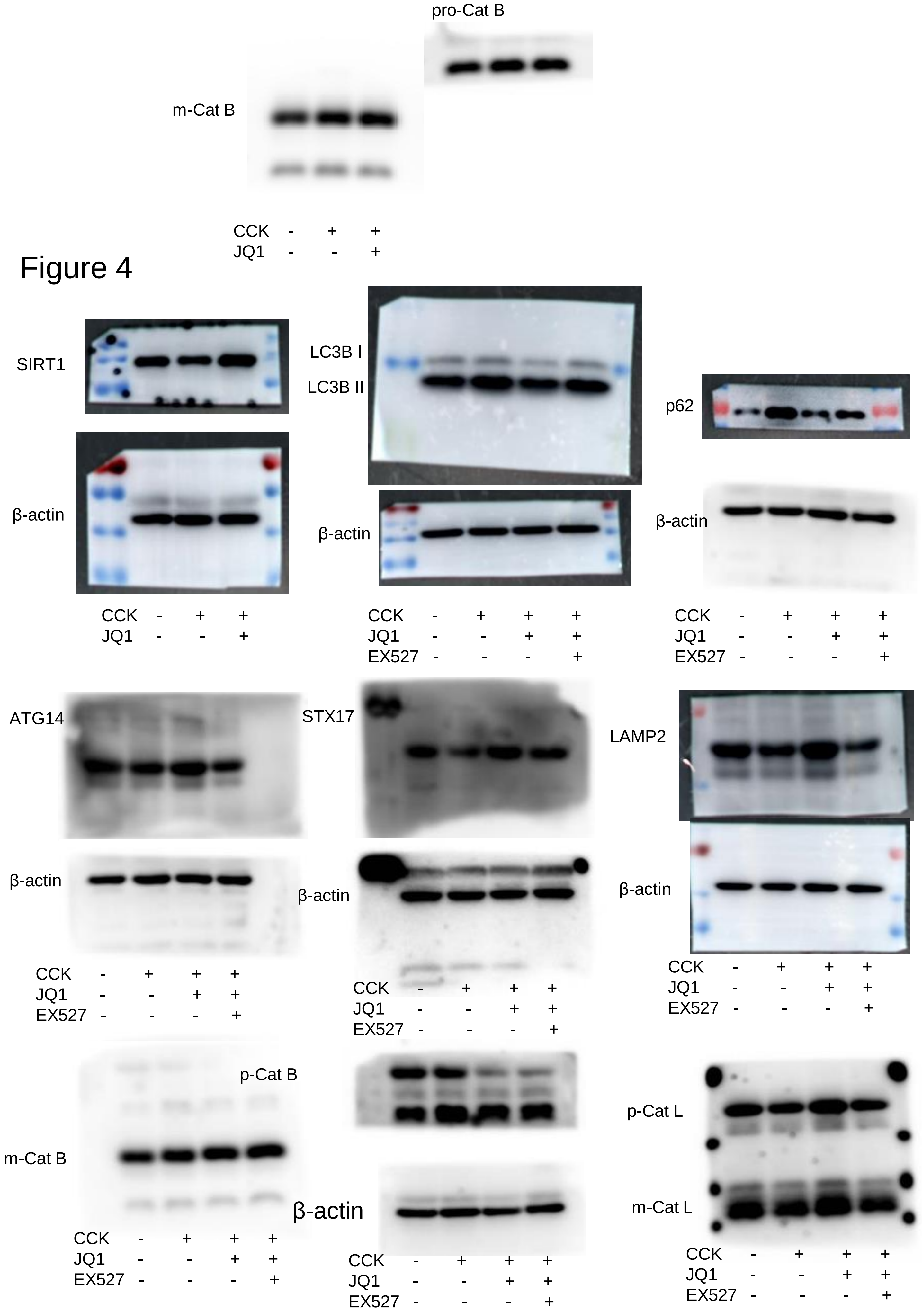
pro-Cat B
m-Cat B
CCK - + +
JQ1 - - +
SIRT1
β-actin
CCK - + +
JQ1 - - +
LC3B I
LC3B II
β-actin
CCK - + + +
JQ1 - - + +
EX527 - - - +
p62
β-actin
CCK - + + +
JQ1 - - + +
EX527 - - - +
ATG14
β-actin
CCK - + + +
JQ1 - - + +
EX527 - - - +
STX17
β-actin
CCK - + + +
JQ1 - - + +
EX527 - - - +
LAMP2
β-actin
CCK - + + +
JQ1 - - + +
EX527 - - - +
p-Cat B
m-Cat B
CCK - + + +
JQ1 - - + +
EX527 - - - +
β-actin
CCK - + + +
JQ1 - - + +
EX527 - - - +
p-Cat L
m-Cat L
CCK - + + +
JQ1 - - + +
EX527 - - - +

Figure 6

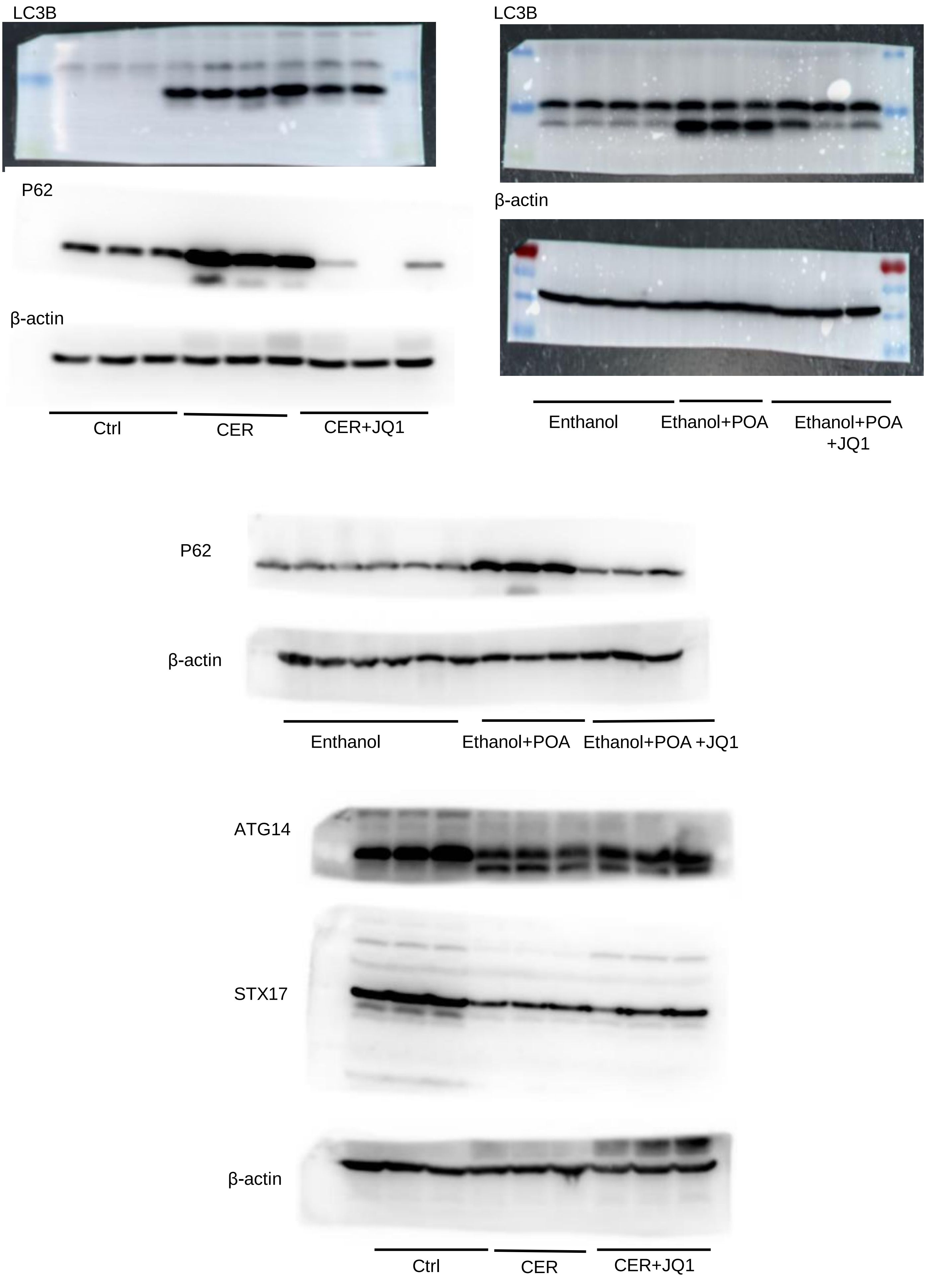

LC3B
P62
β-actin
Ctrl
CER
CER+JQ1
LC3B
β-actin
Enthanol
Ethanol+POA
Ethanol+POA +JQ1
P62
β-actin
Enthanol
Ethanol+POA
Ethanol+POA +JQ1
ATG14
STX17
β-actin
Ctrl
CER
CER+JQ1

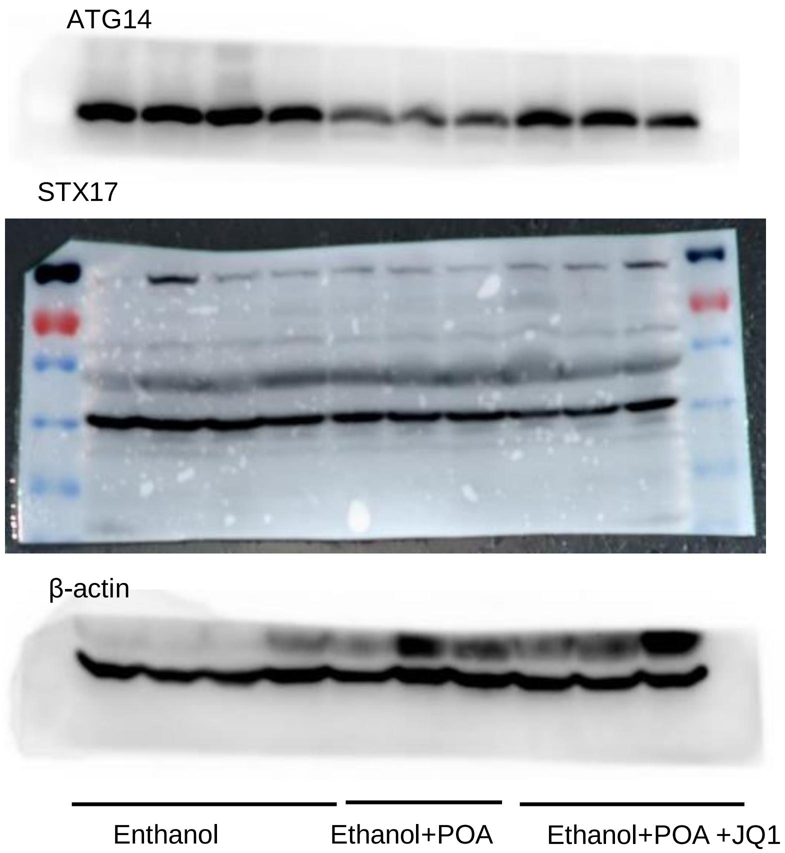
ATG14
STX17
β-actin
Enthanol
Ethanol+POA
Ethanol+POA +JQ1

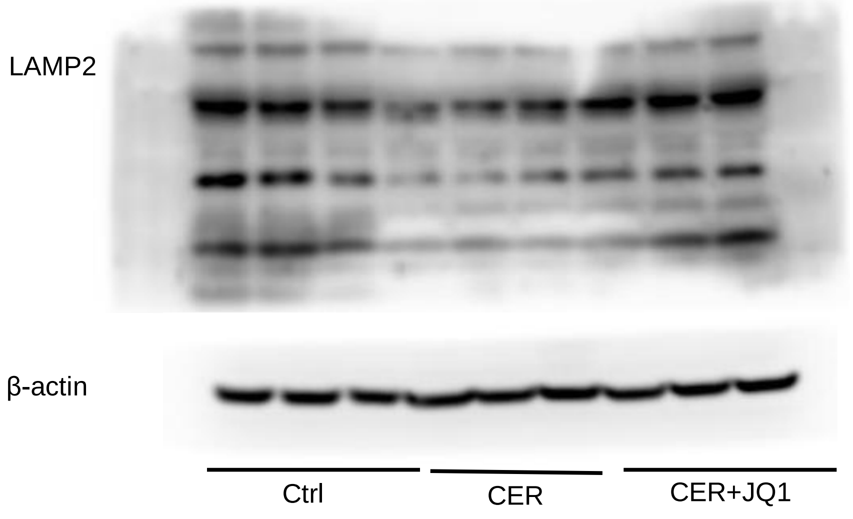
LAMP2
β-actin
Ctrl
CER
CER+JQ1

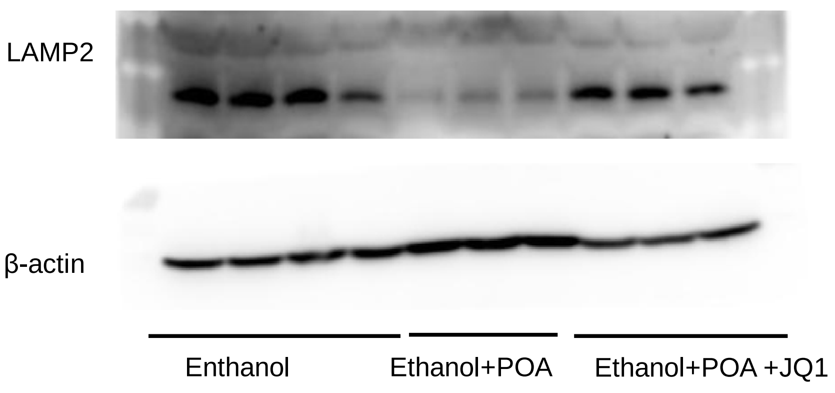
LAMP2
β-actin
Enthanol
Ethanol+POA
Ethanol+POA +JQ1

Supplementary Figure Full Scans of WB

S1

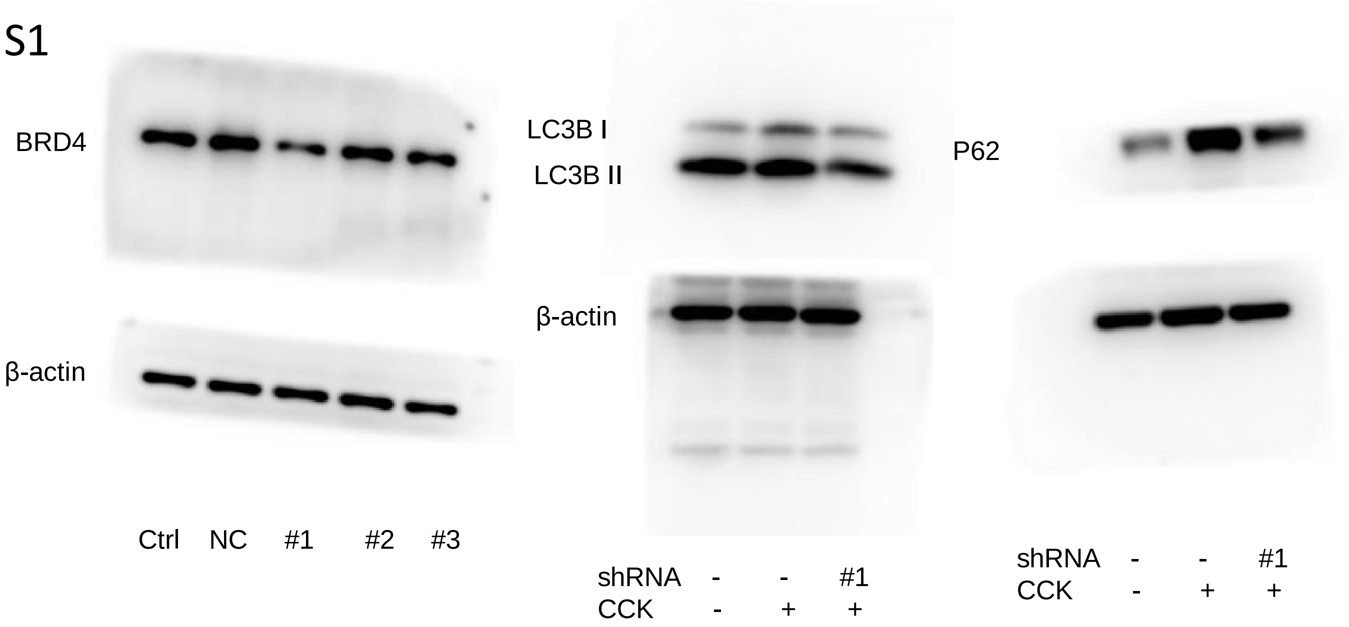

S2

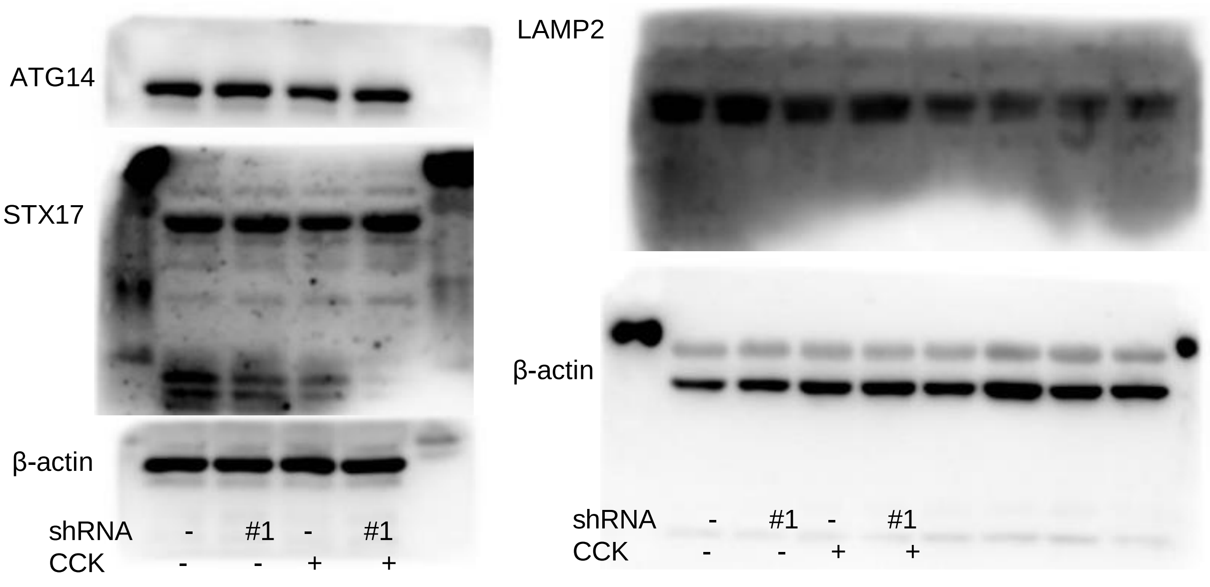

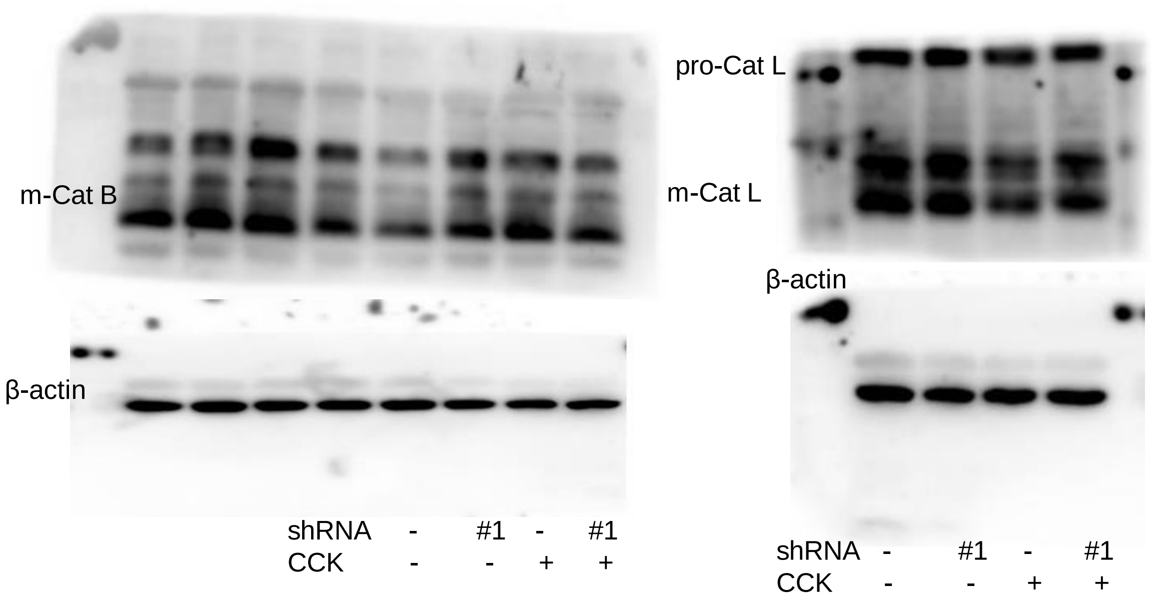
m-Cat B
β-actin
shRNA - #1 - #1
CCK - - + +
pro-Cat L
m-Cat L
β-actin
shRNA - #1 - #1
CCK - - + +

S4

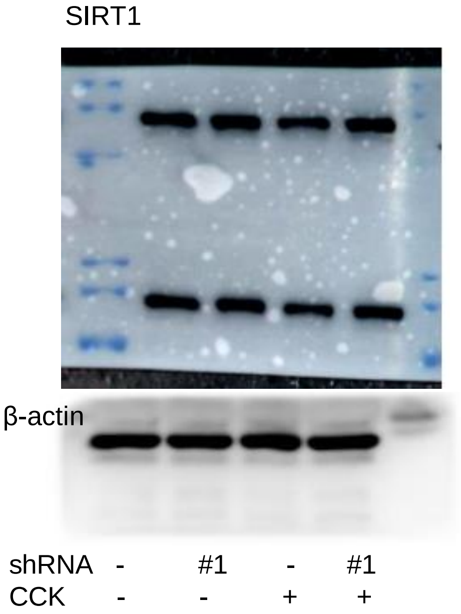
SIRT1
β-actin
shRNA - #1 - #1
CCK - - + +

S5

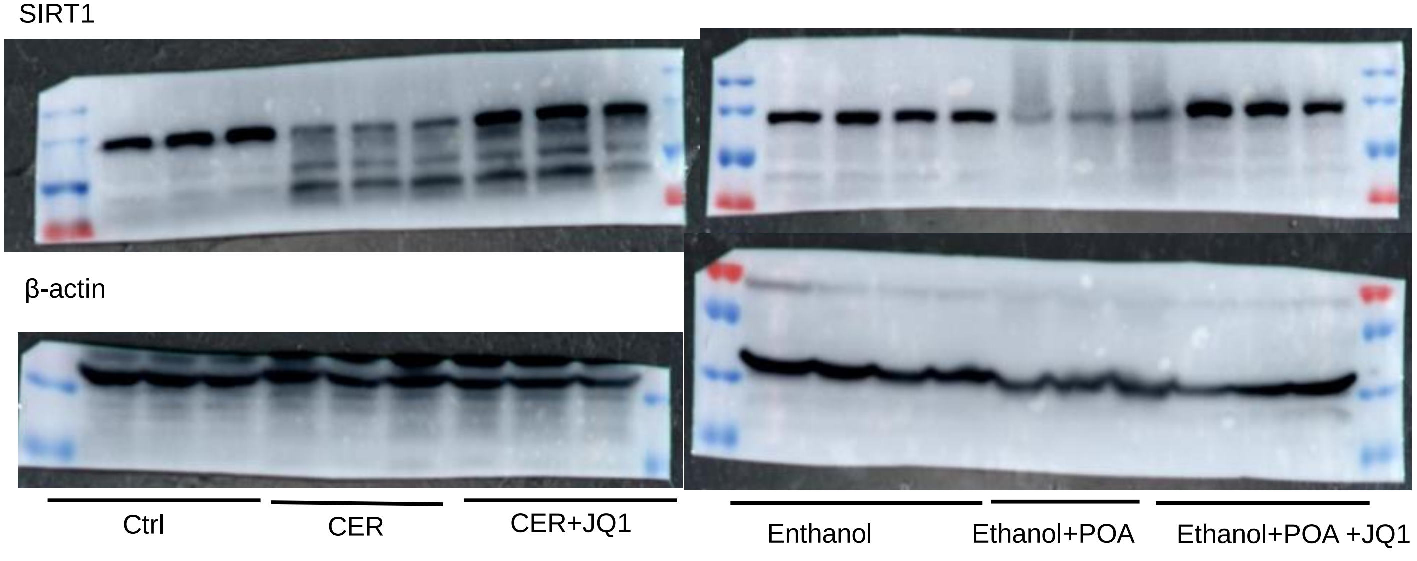
SIRT1
β-actin
Ctrl
CER
CER+JQ1
Enthanol
Ethanol+POA
Ethanol+POA +JQ1
