# Supplementary figures and images for: BRD4 Inhibition Protects Against Acute Pancreatitis Through Restoring Impaired Autophagic Flux
Source: Front Pharmacol. 2020 May 8;11:618. doi: 10.3389/fphar.2020.00618 (PMC7227015; doi:10.3389/fphar.2020.00618)

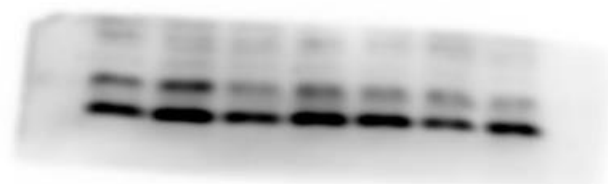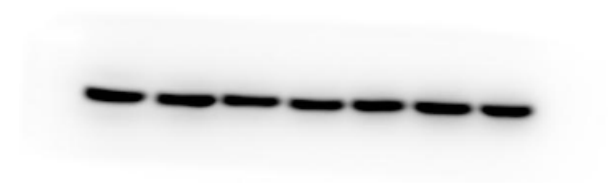

Supplement: Supplementary file 3 [file DataSheet_3.pdf]
